# Supplementary material for: Ultra-rapid detection of nuclear protein of severe fever with thrombocytopenia syndrome virus by colloidal gold immunochromatography assay
Source: PeerJ. 2024 Oct 14;12:e18275. doi: 10.7717/peerj.18275 (PMC11485053; doi:10.7717/peerj.18275)
Supplement: Supplemental Information 1 [file peerj-12-18275-s001.pdf]

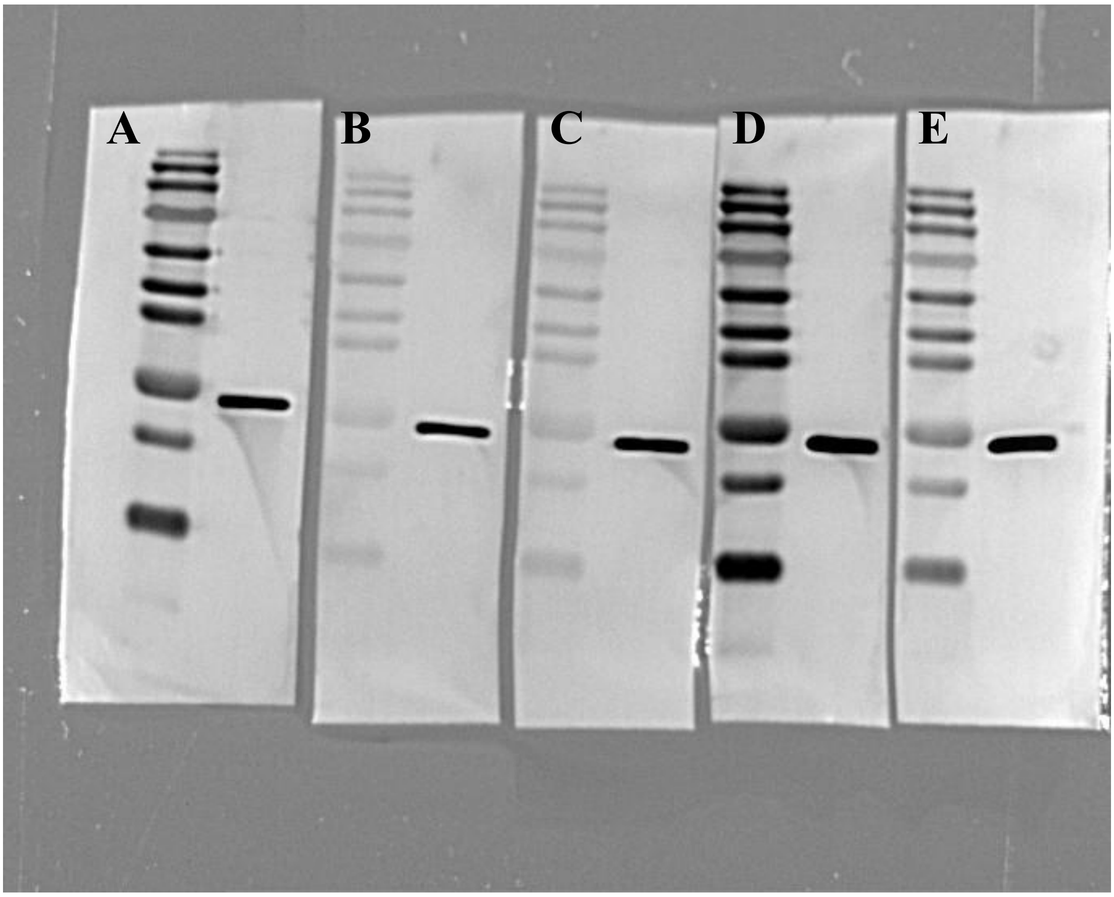

Figure S1: Original Western Blot image of monoclonal antibody against SFTSV-NP protein. (A) The Western Blot image of SV01-13; (E) The Western Blot image of SV06-21.

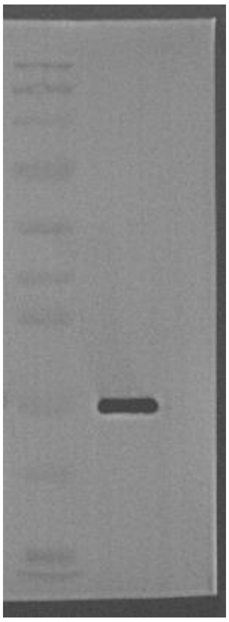

Figure S2: Original Western Blot image of Recombinant SFTSV-NP protein.
