# Supplementary material for: Ultra-rapid detection of nuclear protein of severe fever with thrombocytopenia syndrome virus by colloidal gold immunochromatography assay
Source: PeerJ. 2024 Oct 14;12:e18275. doi: 10.7717/peerj.18275 (PMC11485053; doi:10.7717/peerj.18275)
Supplement: Supplemental Information 2 [file peerj-12-18275-s002.pdf]

The sequence of SFTSV-NP:

5’-GGGAATTCCATATGATGAGTGAATGGTCAAGGATAGCTGTAGAGTTTGGCGAGCAGC
AGCTGAACCTGACCGAATTGGAGGACTTCGCCCGTGAACTGGCGTATGAAGGTCTGGA
CCCGGCGCTCATCATCAAAAAACTGAAAGAGACTGGTGGCGACGACTGGGTTAAAGA
TACCAAATTCATTATCGTGTTTGCGTTGACGAGAGGTAATAAAATCGTCAAGGCTTCCG
GTAAAATGAGCAATTCTGGCAGCAAGCGCCTTATGGCGTTGCAAGAAAAATACGGCCT
GGTGGAGCGCGCGGAAACCCGTTTATCCATTACCCCGGTACGCGTGGCCCAAAGCTTG
CCGACCTGGACGTGCGCTGCGGCTGCGGCGCTGAAGGAGTACTTGCCGGTGGGTCCG
GCGGTTATGAACCTTAAGGTGGAGAACTATCCGCCAGAAATGATGTGTATGGCATTCGG
CTCACTGATCCCGACTGCCGGTGTTAGCGAAGCTACCACGAAGACCCTGATGGAAGCA
TACTCCCTGTGGCAGGACGCTTTCACCAAGACCATTAACGTGAAGATGCGTGGCGCGT
CTAAGACCGAGGTTTATAACAGCTTTCGTGATCCGCTGCATGCAGCCGTTAATTCGGTG
TTTTTTCCGAATGATGTTCGTGTCAAGTGGCTGAAGGCAAAAGGTATTCTGGGCCCAGA
TGGTGTTCCGAGCCGTGCAGCGGAGGTGGCTGCGGCTGCATACCGCAACCTGCTCGAG
ATATTT-3’

SFTSV-39-S:

ACACAAAGACCCCCTTCATTTGGAAACCATGTCACTGAGCAAATGCTCCaACGTTGAC
CTCAAATCTGTTGCAATGAATGCCAACACCGTCAGGCTTGAGCCATCTCTAGGAGAGT
ACCCCACTCTTAGGAGGGACCTCGTTGAATGCTCTTGCAGTGTGTTGACTCTGTCAATG
GTCAAGAGGATGGGTAAAATGACCAACACTGTATGGTTGTTTGGCAACCCGAAAAATC
CTCTTCACCAGCTTGAGCCTGGGCTTGAGCAGCTATTGGACATGTACTACAAGGACATG
AGGTGCTACTCCCAGAGAGAGCTGAGTGCTCTTAGGTGGCCTAGTGGAAAGCCATCTG
TATGGTTCTTACAGGCAGCTCATATGTTCTTCTCCATCAAGAACAGCTGGGCAATGGAA
ACCGGGAGAGAGAACTGGCGGGGCCTCTTCCACAGGATAACAAAAGGCCAGAAGTAT
CTTTTTGAAGGGGACATGATATTGGATTCTCTTGAAGCCATAGAGAAGCGAAGGCTTAG
ACTTGGGTTACCTGAGATCCTAATAACTGGACTATCTCCAATTCTGGATGTGGCcCTCCT
CCAGATAGAGTCACTTGCAAGGCTAAGAGGCATGAGCTTGAACCACCACTTATTCACT
TCTTCCTCATTGCGTAAGCCTCTGTTAGACTGTTGGGACTTCTTTATCCCTATCCGCAAA
AAGAAGACAGATGGCTCATATAGTGTCTTGGATGAGGATGATGAGCCTGGAGTCCTTC
AAGGTTACCCATATCTGATGGCACACTATTTAAACAGGTGCCCATTTCACAACCTCATC
AGGTATGATGAGGAGCTGAGAACTGCAGCCCTGAACACCATCTGGGGAAGAGATTGG
CCAGCCATTGGTGACCCCCCGAAGGAGGTCTAATTTTGTCGAATTGGATCATGAAATTT
AGCCTAATTGGACATGTCAAATTGCTGCTTACAGGTTTCTGTAAGCAGCAGCAGCAAC
CTCAGCAGCTCTGCTGGGGACCCCATCTGGGCCAAGGATTCCCTTGGCCTTCAGCCAC
TTCACCCGAACATCATTGGGAAAGAAGACGGAGTTCACAGCAGCATGGAGAGGATCC
CTGAAGGAGTTGTAAACTTCTGTCTTGCTGGCTCCGCGCATCTTCACATTGATAGTCTT
GGTGAAGGCATCTTGCCACAGAGAGTAGGCCTCCATCAGGGTCTTGGTTGTGGCTTCA
GATACCCCCGCAGTTGGAATCAGGGACCCAAAGGCCATGCACATCATCTCAGGGGGAT
AATTCTCGACCTTCAGGTTCATGACGGCTGGCCCCACTGGGAGATACTCCTTTAAGGCT
GCTGCAGCAGCACATGTCCAAGTGGGAAGGCTCTGCGCTACCCTCACAGGAGTGATTG
AGAGCCTGGTCTCTGCCCTCTCAACCAGTCCATATTTCTCCTGGAGTGCCATCAACCTC
TTAGACCCTGAGTGTGACATTTTCCCTGATGCCTTGACGATCTTATTACCTCGAGTCAG
GGCAAAGACAATGATGAACTTAGTATCCTTCACCCAATCATCTCCACCTGTCTCCTTCA

GCTTCTTGATGATCAAAGCAGGGTCAAGGCCTTCATAGGCTAGCTCTCTCGCGAAATCC
TCAAGCTCAGTCAAATTGAGCTGCTGCTCACCAAACTCCACTGCAATCCTGGACCACT
CTGACATGATCACTCCTTTGCGTCTTTCCTTTTTTGGGGGTCTTTGTGT

SFTSV-39-M:

NTCTACACAGAGACGGCCAACAATGATGAAAGTCATCTGGTtCTcCTCTCTGATCTGCTT
TGTCATTCAATGCAGTGGGGACTCGGGCCCAATCATCTGCGCAGGACCCATTCACTCA
AACAAGAGTGCTGACATACCCCACCTGCTTGGTTACTCTGAGAAGATTTGTCAGATAGA
TCGGCTGATACATGTTTCGTCATGGCTCAGAAACCACTCACAATTTCAGGGCTACGTAG
GGCAGCGAGGTGGACGCTCTCAGGTGAGCTACTACCCAGCTGAAAATTCTTACTCAAG
GTGGAGTGGACTTCTAAGCCCCTGTGATGCTGATTGGCTTGGGATGCTTGTCGTGAAG
AAGGCCAAGGGGTCTGATATGATAGTTCCTGGACCTTCATACAAGGGGAAAGTCTTTTT
TGAACGGCCAACTTTTGATGGATATGTAGGCTGGGGCTGTGGCAGTGGGAAGTCTAGG
ACTGAGTCAGGAGAGCTCTGCAGTTCAGACTCAGGGACTAGTTCTGGTCTTCTACCCT
CAGATAGGGTTCTCTGGATAGGTGATGTTGCTTGTCAACCTATGACACCTATCCCTGAG
GAGACATTCCTGGAGCTGAAGAGCTTTAGCCAAAGTGAATTCCCAGACATATGTAAAA
TTGATGGCATTGTGTTCAACCAGTGTGAGGGTGAGAGTCTACCTCAGCCCTTTGATGTT
GCATGGATGGATGTTGGCCACTCTCACAAAATCATCATGAGGGAGCACAAGACCAAAT
GGGTACAAGAGAGCTCATCCAAGGATTTTGTGTGCTACAAGGAAGGGACTGGGCCTTG
TTCAGAATCAGAAGAAAAGACTTGCAAGACCAGTGGGTCATGCAGGGGGGACATGCA
GTTTTGCAAGGTGGCTGGATGTGAACATGGGGAAGAGGCATCTGAAGCCAAGTGTAGA
TGCTCACTTGTGCACAAGCCCGGGGAAGTCGTTGTGTCTTATGGAGGGATGCGTGTCA
GACCAAAGTGCTATGGATTCTCTAGAATGATGGCAACACTGGAGGTAAACCCACCAGA
GCAAAGGATTGGTCAATGCACTGGCTGCCATCTAGAATGCATAAAAGGGGGTGTGAGG
CTAATCACCCTAACGAGTGAGCTCAAGTCAGCTACTGTCTGTGCTTCCCACTTTTGTAG
TTCTGCCACAAGTGGTAAGAAAAGCACGGAAATTCAATTCCACTCAGGATCATTGGTT
GGGAAAACAGCGGTACACGTCAAAGGGGCATTGGTAGATGGGACTGAATTCACATTTG
AGGGTAGTTGCATGTTCCCAGATGGTTGCGATGCAGTGGACTGCACATTCTGTCGTGA
GTTTCTAAAAAATCCACAGTGCTACCCTGCAAAGAAGTGGCTGTTTATCATTATTGTCA
TCCTCCTTGGATATGCAGGCCTCATGCTACTCACCAATGTCCTCAAGGCAATTGGGGTT
TGGGGGTCATGGGTCATAGCTCCAGTGAAGCTACTGTTTGCCATCATAAAGAAACTGAT
GAGAACTGTAAGCTGCTTGATGGGGAAATTGATGGATAGGGGAAGGCAAGTGATCCAT
GAGGAAATAGGGGAGAATAGAGAGGGCAACCAAGATGATGTTAGGATCGAGATGGCA
AGACCCAGAAGGGTAAGGCATTGGATGTACTCACCTGTCATCCTGACTATTCTAGCAAT
AGGGCTTGCTGAGGGCTGTGATGAGATGGTCCATGCTGATTCTAAACTTGTTTCATGCA
GGCAAGGGAGCGGAAATATGAAGGAATGTGTCACAACTGGGAGGGCGCTTCTTCCTG
CAGTGAACCCAGGTCAAGAGGCATGTCTGCACTTCACGGTACCTGGGAGTCCGGACTC
AAAATGTCTCAAAATTAAGGTAAAGAGGATCAACCTAAAATGTAAGAAGTCATCATCAT
ATTTTGTTCCTGATGCCCGGTCTAGGTGTACATCAGTGAGGAGATGTCGTTGGGCAGGA
GACTGCCAGTCTGGGTGTCCCCCTCATTTCACATCCAACTCCTTTTCTGATGATTGGGC
AGGCAAGATGGACAGGGCTGGTCTAGGATTCAGTGGCTGCTCTGATGGATGTGGAGGA
GCAGCCTGCGGCTGCTTTAATGCGGCCCCTTCATGCATCTTCTGGAGGAAATGGGTAGA
GAATCCACATGGGATCATCTGGAAAGTATCTCCATGTGCCGCATGGGTCCCATCGGCAG
TTATAGAGCTAACAATGCCCTCAGGGGAGGTGAGGACATTCCACCCCATGAGTGGCAT

CCCCACACAAGTCTTCAAGGGTGTTAGTGTAACTTACTTGGGTTCAGATATGGAGGTGT
CTGGCTTGACAGATCTTTGTGAGATAGAAGAGCTCAAGTCCAAGAAGCTGGCATTAGC
TCCCTGCAACCAGGCTGGCATGGGGGTTGTAGGCAAGGTTGGAGAGATACAGTGCAGT
AGTGAGGAAAGTGCCCGTACCATAAAAAAAGATGGGTGCATATGGAATGCAGACCTTG
TGGGCATAGAGCTACGAGTGGATGACGCTGTGTGCTACTCTAAGATCACTAGTGTGGA
GGCAGTTGCAAACTACTCTGCCATACCCACCACTATTGGGGGGCTGAGGTTTGAGAGA
AGCCATGACAGCCAGGGTAAAATATCTGGTAGCCCCCTAGACATCACAGCTATAAGAG
GATCTTTATCTGTTAATTATAGAGGCCTTCGTCTGAGTCTCTCAGAAATTACTGCTACTT
GCACAGGGGAGGTTACAAATGTGAGTGGGTGTTATTCTTGCATGACAGGCGCCAAAGT
CTCCATCAAATTACACAGCAGCAAAAATAGCACTGCCCATGTAAGATGCAAAGGGGAT
GAGACTGCATTCAGTGTCTTGGAGGGGGTCCATAGCTACACTGTCAGTCTCAGTTTTG
ACCATGCAGTAGTCGATGAGCAGTGCCAACTGAACTGTGGGGGACATGAGAGCCAAG
TGACTCTAAAAGGTAACCTCATCTTCCTGGATGTCCCAAAATTTGTGGATGGCAGCTAC
ATGCAGACATATCACAGTACTGTGCCCACAGGGGCAAATATCCCAAGCCCTACAGATTG
GCTGAATGCCCTGTTTGGCAATGGGCTGAGTAGGTGGATTCTGGGGGTGATAGGGGTT
CTACTTGGGGGATTGGCTCTCTTTTTCTTGATTATGTCTTTGTTCAAGCTGGGAACAAA
ACAGGTATTTCGATCAAGGACGAAGCTGGCTTAGATGGGCAAAATTCTGGTCTAATGA
CCCTCTGGGAGCAGTGCTCTCAGGGGAGTTGGCTCACAGTGTGCATGTTTCGTAGTTC
CGGCTCACATTCTTAGAACATGGAGGTTCTATTGAAGTGTTGGCCGGTCTTTGTGT

SFTSV-39-L:

NNNTCTACACAGAGACGCCCAGATGAACTTGGAAGTGCTTTGTGGTAGGaTAAaCGTG
GAAaATGGGCTGTCTCTTGGAGAACCAGGCCTGTACGACCAAATCTACGACAGGCCAG
GGCTACCAGACCTAGATGTGACTGTCGATGCCACAGGTGTGACGGTGGACATAGGGGC
TGTGCCAGACTCAGCATCACAACTGGGTTCATCAATCAATGCTGGGTTGATCACAATTC
AGCTCTCTGAAGCATATAAGATCAATCATGACTTCACGTTCTCTGGTCTGTCAAAGACT
ACAGACCGACGCCTCTCAGAGGTATTCCCCATTACCCATGATGGTTCTGATGGGATGAC
CCCTGATGTGATTCACACCAGATTGGATGGAACCATTGTGGTGGTTGAATTCTCAACCA
CTAGGAGCCATAACATTGGGGGCCTGGAGGCAGCATACAGGACAAAGATAGAAAAATA
TAGGGACCCAATCTCAAGGCGTGTTGATATCATGGAGAACCCGAGGGTCTTCTTTGGC
GTTATTGTAGTCTCGTCAGGAGGGGTTCTGTCCAACATGCCCCTAACCCAGGATGAGGC
AGAGGAGCTTATGTACAGGTTCTGCATAGCCAATGAGATCTACACTAAGGCTAGATCTA
TGGATGCGGACATTGAGCTACAGAAGAGTGAAGAGGAGCTTGAAGCCATTAGCAGGG
CACTATCATTCTTCAGTTTGTTTGAGCCTAACATTGAAAGAGTGGAAGGAACATTCCCT
AATTCAAAGATCGAGATGCTGGAACAGTTTCTCTCAACACCAGCTGATGTTGACTTCAT
CACCAAGACCCTCAAAGCTAAAGAGGTGGAGGCCTATGCTGATCTTTGTGACAGCCAC
TACCTAAAGCCTGAGAAAACCATTCAAGAGCGGCTGGAAATCAATAGATGTGAGGCTA
TTGACAAAACTCAGGATCTCCTGGCTGGCCTGCATGCAAGAAGCAACAAGCAAACAT
CATTGAATCGAGGGACAGTCAAACTCCCGCCCTGGCTACCAAAGCCATCAAGTGAGTC
AATAGACATCAAGACCGACTCAGGCTTTGGATCCTTAATGGATCATGGCGCATATGGTG
AACTGTGGGCAAAGTGCCTCCTAGATGTCTCGCTGGGAAATGTGGAGGGGGTAGTAAG
TGACCCTGCAAAAGAACTTGACATTGCCATCTCTGATGATCCAGAAAAAGACACCCCC
AAAGAGGCAAAGATAACCTATAGGCGATTCAAGCCTGCCTTAAGTTCAAGTGCCCGTC
AGGAATTTTCTCTCCAAGGAGTGGAGGGGAAGAAGTGGAAGAGAATGGCAGCAAACC

AGAAGAAAGAAAAAGAGTCCCATGAGACATTGAGCCCTTACCTGGATGTTGAAGACA
TTGGGGATTTCCTAACATTCAACAATCTCCTTGCAGATTCGAGGTATGGAGATGAGTCC
ATCCAGAGAGCTGTGTCAATCTTGTTGGAAAAGGCATCTGCCATGCAAGACACAGAGC
TCACTCATGCCCTCAATGACTCATTTAAGAGGAACCTAAGCAGTAATGTGGTTCAGTGG
TCTCTTTGGGTCTCATGTTTAGCACAGGAGCTAGCTAGTGCCCTGAAGCAGCACTGCA
GGGCTGGTGAGTTCATCATCAAGAAGCTGAAGTTCTGGCCTATCTACGTCATTATCAAG
CCGACCAAGTCATCATCCCACATCTTCTACAGCTTAGGGATCCGCAAGGCTGACGTGA
CAAGGAGGCTAACTGGTAGAGTCTTCTCTGACACCATTGATGCTGGGGAATGGGAGCT
AACAGAGTTCAAAAGCCTGAAGACATGCAAGCTCACGAATCTTGTCAACTTGCCATGC
ACCATGCTGAACTCAaTAGCCTTCTGGAGAGAGAAGTTGGGCGTGGCTCCATGGCTGG
TTCGAAAGCCTTGTTCAGAGCTCAGAGAGCAGGTGGGCCTGACCTTCCTGATCAGTCT
GGAGGACAAGTCTAAGACTGAGGAGATCATCACCTTGACAAGGTACACCCAGATGGA
GGGCTTTGTCTCTCCTCCCATGCTGCCTAAGCCCCAAAAGATGCTAGGGAAACTGGAA
GGACCTTTGAGAACTAAGCTACAGGTATACCTCCTCAGGAAGCATCTAGATTGCATGGT
GCGAATTGCTTCTCAGCCGTTCAGCCTAATCCCCAGAGAGGGAAGGGTTGAGTGGGGA
GGAACATTCCATGCCATCTCAGGTCGGTCCACAAACCTTGAGAATATGGTGAACAGCT
GGTACATTGGGTACTACAAGAACAAAGAGGAGTCAACAGAGCTGAATGCCCTCGGAG
AAATGTATAAGAAGATTGTGGAGATGGAAGAGGACAAGCCCAGCAGCCCTGAGTTTCT
AGGGTGGGGGGACACAGATTCCCCTAAGAAGCATGAGTTCTCACGGAGCTTCCTCAG
AGCTGCTTGCTCATCTCTGGAGAGAGAAATTGCTCAGCGACATGGAAGACAATGgAAG
CAGAACCTTGAGGAGCGTGTCCTGAGAGAGATTGGGACcAAGAACATCCTGGACCTTG
CATCCATGAAGGCCACAAGCAACTTTTCCAAAGACTGGGAGCTCTACTCAGAAGTCCA
GACAAAGGAGTACCATAGGTCCAAACTGCTGGAGAAGATGGCCACATTGATTGAGAA
GGGAGTTATGTGGTACATTGATGCTGTGGGCCAGGCATGGAAGGCAGTTCTAGATGAC
GGGTGCATGCGAATCTGTCTCTTCAAAAAGAATCAGCATGGTGGCCTCAGAGAGATCT
ACGTTATGGATGCGAATGCCCGGCTCGTGCAGTTTGGGGTTGAGACCATGGCTAGGTG
TGTCTGTGAGCTGAGCCCACATGAGACTGTTGCCAACCCTAGGCTCAAGAATTCCATC
ATAGAGAACCATGGGCTGAAGTCAGCCCGTAGCCTTGGCCCTGGCTCTATAAACATAA
ACTCATCCAATGATGCCAAGAAGTGGAATCAGGGGCACTACACAACAAAGCTAGCTCT
AGTTCTTTGTTGGTTTATGCCAACCAAATTCCATAGATTCATTTGGGCTGCCATTTCCAT
GTTCCGGAGGAAAAAGATGATGGTGGACCTAAGGTTTTTGGCTCACCTCAGTTCTAAA
TCTGAGTCCAGGTCAACTGATCCGTTTAGGGAAGCAATGACAGACGCCTTCCATGGTA
ATAGGGAAGTCTCATGGATGGACAAAGGGCGAACTTACATAAAGACAGAGACAGGAA
TGATGCAGGGCATACTGCACTTTACATCCAGTCTCCTCCACTCTTGTGTTCAGAGCTTCT
ACAAGTCTTATTTCGTCTCGAAGCTCAAGGAGGGCTACATGGGGGAAAGCATCAGTGG
GGTGGTGGATGTCATAGAAGGCTCTGACGACTCAGCGATCATGATCAGCATACGCCCTA
AGTCAGATATGGATGAAGTCCGATCAAGGTTTTTTGTTGCTAACTTGCTCCACTCTGTC
AAGTTCTTGAACCCTTTGTTTGGAATTTATTCATCAGAGAAATCAACAGTGAACACAGT
GTATTGTGTCGAGTATAACTCTGAATTCCATTTCCATAGGCACTTGGTTAGACCCACACT
GAGATGGATTGCAGCGTCTCACCAAATCTCAGAGACTGAAGCCCTTGCAAGCAGGCA
AGAGGATTACTCCAACCTTCTAACCCAGTGCTTGGAAGGAGGGGCCTCATTCTCTCTTA
CGTACCTCATACAGTGCGCTCAGCTCCTGCACCACTACATGCTTCTAGGGCTATGCTTA
CATCCCTTGTTTGGAACATTCATGGGGATGCTGATATCAGACCCAGATCCTGCCTTAGG
GTTCTTCCTCATGGACAACCCTGCATTCGCAGGGGGAGCAGGATTTAGATTCAATCTGT

GGAGAGCCTGCAAGACTACAGACCTTGGGCGGAAGTATGCATATTATTTAATGAGATACAGGGTAAAACAAAGGGAGATGAGGACTACAGAGCTCTAGATGCCACATCGGGAGGAACTCTCAGCCACTCTGTTATGGTGTACTGGGGGGACAGGAAGAAGTATCAGGCCTTATTGAGCAGAATGGGCCTTCCTGAAGACTGGGTGGAGCAGATAGATGAGAATCCTGGAGTCCTTTACAGGAGAGCTGCCAACAAGAAGGAACTACTCCTAAAACTGGCGGAGAAGGTTCATTCACCTGGTGTGACTAGCAGCCTGAGCAAAGGGCATGTAGTGCCTCGGGTGGTGGCAGCAGGAGTATACCTTCTCTCACGCCaCTGCTTTCGCTTTAGTTCAAGCATCCATGGCAGGGGCTCAACACAGAAGGCTAGCCTCATAAAACTGCTGATGATGTCTTCTATTTCTGCCATGAAGCATGGGGGCTCACTAAACCCTAACCAGGAGCGAATGCTCTTCCCTCAGGCCCAAGAGTATGACAGAGTTTGCACATTGCTTGAGGAAGTTGAACACCTATCAGGGAAATTTGTTGTTAGGGAGAGGAACATTGTCAGGAGCCGCATAGACTTGTTCCAAGAGCCAGTGGACTTGCGGTGCAAGGCAGAAGATCTGGTGTCAGAGGTGTGGTTTGGCCTGAAAAGGACTAAGCTTGGACCCCGTCTCCTCAAGGAAGAGTGGGACAAGCTTAGGGCCTCTTTTGCATGGCTGAGCACAGACCCATCTGAAACATTGAGGGATGGTCCTTTTCTTAGTCATGTGCAGTTTAGGAACTTCATAGCCCACGTTGATGCCAAATCAAGGTCAGTCAGGCTCCTAGGTGCCCCCGTGAAGAAGTCAGGTGGGGTCACCACTATAAGCCAAGTAGTCAGAATGAACTTCTTCCCTGGTTTTAGCCTAGAAGCTGAGAAGAGCTTAGACAATCAGGAAAGACTTGAGAGCATCTCCATCCTCAAGCATGTCTTGTTCATGGTCTTAAATGGCCCATACACTGAGGAGTACAAGCTAGAAATGATCATAGAGGCCTTTTCCACTCTTGTGATACCTCAACCATCAGAGGTCATCAGGAAATCAAGGACCATGACTCTATGCCTCTTGTCGAATTACTTGTCTAGTAGGGGTGGTTCCATTCTAGACCAGATTGAGAGGGCACAGTCAGGCACTCTAGGAGGCTTCAGCAAGCCCCAGAAGACATTCATTAGGCCAGGAGGTGGTGTTGGCTACAAGGGAAAAGGTGTGTGGACTGGAGTGATGGAGGACACCCATGTTCAAATTCTGATAGATGGAGATGGGACTAGTAACTGGCTTGAGGAGATCAGGCTCAGTAGTGATGCCAGGCTTTATGATGTTATTGAATCCATCCGAAGGTTATGTGATGACCTTGGGATCAACAACAGGGTGGCATCTGCATATAGAGGTCATTGCATGGTTAGGCTGAGTGGATTCAAGATCAAGCCAGCATCAAGGACTGACGGCTGTCCAGTCAGGATTATGGAAAGGGGCTTCAGGATTAGGGAACTTCAGAACCCAGATGAGGTCAAGATGAGAGTGAGGGGCGACATCCTCAACCTCTCTGTCACCATACAAGAGGGAAGGGTCATGAACATTCTAAGCTACAGGCCGAGAGACACTGATATATCAGAGTCAGCTGCAGCATATCTCTGGAGCAATCGAGACCTCTTCTCCTTTGGGAAGAAGGAGCCATCCTGCAGCTGGATCTGCTTGAAAACTCTTGACAATTGGGCCTGGTCACATGCCTCAGTTCTCCTGGCAAATGATAGGAAGACCCAAGGCATTGACAATAGAGCCATGGGGAATATTTTCAGGGACTGTCTCGAGGGTTCTCTTAGGAAGCAGGGACTGATGAGGTCAAAGCTCACAGAGATGGTGGAGAAGAATGTAGTTCCTTTAACAACTCAAGAGCTCGTCGACATCTTGGAGGAGGACATTGACTTTTCAGATGTCATAGCTGTGGAGCTCTCAGAGGGGTCACTTGACATTGAATCCATCTTTGACGGGGCGCCTATCTTGTGGTCTGCAGAGGTGGAAGAGTTTGGAGAAGGAGTGGTGGCTGTGAGCTATTCCAGTAAGTACTATCATCTAACCCTGATGGACCAGGCTGCTATCACAATGTGTGCGATCATGGGTAAGGAAGGCTGTAGAGGGCTCCTCACTGAGAAGAGATGCATGGCAGCCATACGAGAGCAGGTGCGGCCATTCCTCATATTCCTGCAAATTCCTGAGGACAGCATTTCTTGGGTGTCTGATCAATTCTGCGACTCCAGGGGTCTTGATGAAGAGAGCACCATTATGTGGGGTTAACTTTAAAACACGGTTGGCACGCAGTTGATGTATCTGTGGGTAACTAGGGAATGTAGGTTTTAGAAGGATTTCCTTAAGATCTGGGCGGTCTTTGTGT
